# Routine upper gastro-intestinal tract endoscopy before elective cholecystectomy for symptomatic gallstones-justified

**DOI:** 10.1038/s41598-024-64019-2

**Published:** 2024-06-18

**Authors:** Sherwyn Morrison, Taole Mokoena

**Affiliations:** Department of Surgery, Faculty of Health Sciences, University of Pretoria, Private Bag X323, Pretoria, 0001 South Africa

**Keywords:** Gallstones, Routine, Upper gastrointestinal endoscopy, Cholecystectomy, Oesophagogastroscopy, Gall bladder disease

## Abstract

Gallstones are common in Western countries and increasing in developing countries through adoption of western lifestyle. Gallstones may cause life-threatening complications, including acute cholecystitis, acute cholangitis, and acute pancreatitis. Cholecystectomy is the treatment of choice for symptomatic gallstones. Presentation of symptomatic gallstones may be indistinguishable from that of other upper gastro-intestinal tract (UGI) pathologies. Some surgeons routinely perform preoperative UGI endoscopy to diagnose and treat concomitant UGI pathology. A prospective cross-sectional observational study was undertaken at University of Pretoria teaching hospitals to evaluate this practice. Patients aged 18 years and older, with symptomatic gallstones but did not satisfy Tokyo guidelines for acute cholecystitis were recruited. UGI endoscopy was performed before cholecystectomy. There were 124 patients, 110 (88.7%) females and 14 (11.3%) males, mean age 44.0 (13.2) (range: 22–78) years. Most common symptoms were right upper quadrant (RUQ) pain (87%), epigastric pain (59.7%), nausea (58.1%) and vomiting (47.9%). Clinically, 80% had RUQ tenderness and 52.4% epigastric tenderness. UGI endoscopy found 35.4% pathology, 28.2% were active, and comprised acute gastritis (27.4%), peptic ulcers (4.8%), duodenitis (3.2%) and oesophagitis (2.4%). Twelve patients had more than one pathology. This warranted treatment before elective cholecystectomy and justifies the practice of routine preoperative UGI endoscopy.

## Introduction

The incidence of gallstones is up to 20% worldwide and very common in Western countries partly because of diet which is associated with cholesterol gallstones^1^. It is increasing in developing countries including among black patients in South Africa^2,3^. A majority of gallstones remain quiescent and only about 20% become symptomatic^4^. The most common symptom of gallbladder gallstones or cholecystolithiasis is biliary colic but a significant number may result in serious complications including acute cholecystitis, acute cholangitis, acute pancreatitis and cholangiocarcinoma^4,5^.

Symptoms of gallstone disease may be similar to those of other upper gastro-intestinal tract (UGI) pathologies such as gastritis, oesophagitis, peptic ulcers and gastro–esophageal reflux disease. A number of apparently symptomatic gallstones patients may be afflicted by another UGI pathology which may warrant specific treatment^6–8^. It is therefore logical that patients with symptomatic gallstones who do not fully satisfy the Tokyo guidelines for acute cholecystitis diagnosis^9^ should have other UGI pathology excluded before elective cholecystectomy^10,11^. However, a few authors refute the necessity for UGI endoscopy before cholecystectomy^12^. It has been our policy to perform UGI endoscopy on patients with presumed symptomatic gallstones who do not satisfy the Tokyo guidelines for acute cholecystitis diagnosis before elective cholecystectomy for cholecystolithiasis.

Herein we report our prospective evaluation of this practice.

## Patients and methods

A prospective cross-sectional observational study was undertaken at the University of Pretoria teaching hospitals, Steve Biko Academic Hospital and Kalafong Tertiary Hospital.

Patients with cholecystolithiasis aged 18 years and older who had presumed symptomatic gallstone disease and did not fully satisfy the Tokyo guidelines for acute cholecystitis diagnosis were recruited for the study from December 2016 to July 2019. Thus, patients with asymptomatic gallbladder stones and definite acute cholecystitis were excluded.

All patients had thorough history taken, a full physical examination, and relevant blood tests done, which included white cell count (WCC), C-reactive protein (CRP), liver function tests and serum lipase. Abdominal ultrasonography for gallstones was performed as part of the diagnostic work up. Patients were subjected to UGI endoscopy before elective cholecystectomy and those with UGI pathology were appropriately treated pre-operatively.

### Statistics consideration

A biostatistician determined that a minimum of 62 patients would be required to reliably predict that 20% of patients would have a concurrent UGI pathology at 95% confidence interval. Nominal statistics were expressed as percentages and proportions.

### Ethical considerations

The study was approved by the Research Ethics Committee of the University of Pretoria, Faculty of Health Sciences (reference 386/2016) and conducted according to the Helsinki Declaration on research on human subjects. Informed consent to participate in the study was obtained from all patients.

## Results

One hundred and twenty four patients were enrolled in the study. Patient demographic details are shown in Table 1. It is noted that the mean age was 44.0 years, a majority were female (88.7%) and black Africans (55.6%). The predominant symptoms were right upper quadrant (RUQ) (87%) or epigastric (59.7%) pain and major physical signs were RUQ (80.6%) or epigastric (52.4%) tenderness.

**Table 1:** Clinical and upper gastro-intestinal tract endoscopy findings in patients with presumed symptomatic gallstones.

Total number of patients: 124
Patient demographics Gender: females 110 (88.7%); males 14 (11.3%) Race: African 69 (55.6%); White 50 (40.3%); Coloured 2 (1.6%); Indian 3 (2.4%) Age: mean 44.0 (13.2) years (range 22–78)
Selected symptoms and signs RUQ ^#^pain: 109 (87%) Epigastric pain: 74 (59.7%) Nausea: 72 (58.1%) Vomiting: 59 (47.6%) Fever: 16 (12.9%) RUQ tenderness: 100 (80.6%) Epigastric tenderness: 65 (52.4%)
Endoscopy findings* Normal: 89 (71.8%) Abnormal: 35 (28.2%) Acute gastritis: 34 (27.4%) Peptic ulcer: 6 (4.8%) Duodenitis: 4 (3.2%) Oesophagitis 3 (2.4%) Hiatus hernia: 12 (9.7%)

^#^RUQ = right upper quadrant.

*12 patients had more than one pathological endoscopy finding.

All patients had gallbladder stones on ultrasonography but no sonographic signs of acute cholecystitis, these being the recruitment criteria for the study. Mean WCC was 9.13 (3.75) × 10^9^/L (range 1.52–21.4 and median = 8.56), and mean CRP was 42.92 (66.79) IU/L (range 1–313 and median = 9.0).

UGI endoscopy detected active UGI pathology in 28.2% where acute gastritis was most common (27.4%) and peptic ulcer the most serious (4.8%). There were 12 patients with hiatus hernia but only three of them had active pathology. Therefore 44 (35.4%) had abnormal UGI endoscopy findings.

## Discussion

Gallstone disease is a leading cause of elective abdominal surgery^5^. However, a majority of gallstones are quiescent, being discovered incidentally during radiological examinations, particularly ultrasonography, undertaken for other reasons, or during abdominal surgery, or at autopsy^4,5^. Symptomatic gallstones may lead to complications some of which may be life-threatening such as acute cholecystitis, acute cholangitis, acute pancreatitis and cholangiocarcinoma^4,5^. A majority of gallstones are cholesterol stones associated with a western lifestyle and diet. The South African black population is rapidly westernising and as such they show increasing incidence of gallstones^2,3^. Presentation of symptomatic gallstones may be indistinguishable from that of other causes of foregut or UGI pathology^6–8^. Consequently, a number of patients may undergo cholecystectomy, the management of choice for symptomatic gallstone disease^4,5^, while the actual cause of their symptoms is a different UGI pathology^10,11,13,14^. Such patients may continue to experience UGI symptoms after successful and uneventful cholecystectomy as postcholecystectomy syndrome^15–18^. Although there are other possible causes of postcholecystectomy syndrome^19–23^, most concurrent UGI pathology can be readily diagnosed by UGI endoscopy and appropriately treated before elective cholecystectomy^24^. Indeed, cholecystectomy may be cancelled in such cases^10–12,15^. This prospective study found concurrent UGI pathology in 35.4% patients and 28.2% had active pathology. This concurs with most published prevalence reports of 20–46% concurrent UGI pathology. The current study validates our practice of UGI endoscopy and treatment of associated UGI pathology before elective cholecystectomy in a South African hospital patient population which is predominantly black. Indeed, it is the first report of its kind in South Africa or sub-Saharan Africa. It is probable that prior diagnosis and treatment of co-existing UGI pathology would reduce the incidence of postcholecystectomy syndrome. However, this would need to be confirmed by a prospective randomized trial where some patients would undergo UGI endoscopy and treatment of UGI associated pathology before elective cholecystectomy for cholecystolithiasis, while others would not.

## Conclusion

The practice of routine UGI endoscopy before elective cholecystectomy has been justified by the finding of active UGI pathology which warranted treatment in a significant proportion of patients with presumed symptomatic gallstones in an African setting.

## Data Availability

Data is available from the corresponding author on reasonable request.
